# Metal Particle Pencil Beam Spray-Coating Method for High-Density Polymer–Resin Composites: Evaluation of Radiation-Shielding Sheet Properties

**DOI:** 10.3390/ma16186092

**Published:** 2023-09-06

**Authors:** Seon-Chil Kim

**Affiliations:** 1 Department of Biomedical Engineering, Keimyung University, 1095 Dalgubeol-daero, Daegu 42601, Republic of Korea; chil@kmu.ac.kr; Tel.: +82-10-4803-7773; 2 Department of Medical Informatics, School of Medicine, Keimyung University, 1095 Dalgubeol-daero, Daegu 42601, Republic of Korea

**Keywords:** medical radiation, radiation shielding, shielding sheet, tungsten, polyethylene

## Abstract

Medical shielding suits must be lightweight and satisfy the requirements of thin films to guarantee user mobility and safety. The thin film weight is related to the density and thickness, which are associated with the particle dispersion in shielding materials. An even distribution of metal particles in a polymer can maintain the spacing among them. This paper proposes a pencil beam spray-coating method that involves spraying a constant amount of a polyethylene and tungsten mixture in a thin beam onto a nonwoven fabric at a constant speed. This technique yields higher productivity than does the electrospinning method and is expected to produce materials with better shielding performance than that of materials obtained using the calender method. The shielding performance was evaluated by manufacturing shielding sheets (thickness: 0.48–0.54 mm) using the calender and pencil beam spray-coating methods under the same conditions. The densities and performances of the sheets differed significantly. The sheet manufactured using the proposed method had an even particle dispersion and exhibited 2–4% better shielding performance than did that manufactured using the calender method. Therefore, the pencil beam spray-coating method can effectively satisfy the requirements of thin films for medical radiation-shielding materials while increasing the material flexibility.

## 1. Introduction

The dispersion state of shielding materials has a profound influence on the performance of shielding fabrics in medical radiation settings. Theoretically, the greater the amount of shielding material, the superior the shielding performance [1]. However, the marketability of shielding materials is relatively low owing to the economic feasibility and weights of products. Moreover, the reproducibility of the shielding performance cannot be guaranteed because of clumping in the distribution of the shielding material throughout the fabric [2]. Recently, a technology for fabricating radiation shields using thin films was proposed [3]. The thinner the film, the easier the dispersion of the shielding material. The manufacturing technology employed for shielding fabrics evenly disperses metal particles, which are functional shielding materials, to appropriate locations [4]. In previous studies, dispersion relied on chemical actions that increased the affinity among materials during the mixing of polymers and shielding materials [5]. This technology primarily reduced the spacing among particles by reducing the particle size of the shielding material [6].

However, the spacing between particles in a shielding material is irregular, and agglomeration and voids may occur between pairs of particles depending on their bonding state with the polymer [7]. In particular, the dispersion of the shielding material in the polymer, typically affected by the material’s affinity, should occur in the liquid phase to enable the manufacturing of a flexible radiation-shielding fabric for clothing applications [8]. Quantitative prediction of the dispersion of the shielding material is impossible; therefore, an approximate value is estimated from the weight ratio, which is the input amount of shielding material in the process [9].

The same shielding material mass ratio is applied to the process technology as a controlling factor to maintain the reproducibility of the shielding performance; however, predicting the proper dispersion of the shielding material particles in the actual sheet manufacturing process is difficult [10]. To solve this problem, a technique that maintains the gap between the shielding material particles rather than relying on the properties of the polymer can be applied [11]. An interparticle spacing control technology can evenly disperse metal particles by reducing air bubbles or voids and controlling the dispersion of the shielding material based on the particle positional information. The range of the radiation energy intensity used in medical institutions is constant and depends on the body part to which radiation is applied; for radiation levels ≥0.25 mmPb (based on the lead equivalent), >90% of the incident energy can be shielded [12]. A 0.25 mmPb lead equivalent can be shielded with a shielding mixture combined with a polymer of thickness ≥0.5 mm. The thickness can be adjusted according to the degree of dispersion of the metal material, such as tungsten, instead of the base polymer material [13]. Therefore, a novel model for the shielding material dispersion method is necessary to manufacture a medical-radiation-shielding sheet with an adjustable thickness. In this study, a coating method that maintains the distance between neighboring metal particles (mixed with the polymer) and disperses them evenly was devised. A process technology was developed to disperse a mixture of polyethylene (PE) polymer and tungsten metal particles in the liquid state in the form of a pencil beam. The multilayered structure in the shielding fabric of the multicasting method was manufactured to amplify the radiation defense effect. Using tungsten for long-term repetitive coating operations is difficult as it comprises metal particles with high-specific-gravity characteristics [14]. To this end, a few studies developed a model in which PE and tungsten were mixed in the liquid state through electrospinning to form a desired pattern [15,16]. Herein, we focused on enhancing the productivity of the process technology through direct mixing spinning. Research and development of these process technologies are expected to contribute to the production of highly effective shielding products.

Patients, guardians, and medical staff in medical institutions utilize shielding suits, which must be flexible to ensure user mobility [17]. Therefore, metal materials are combined with flexible materials such as polymers to produce sheets or films because they cannot be directly applied to clothing for shielding purposes [18]. In this experiment, a shielding sheet that can satisfy the set flexibility and shielding performance requirements was manufactured and employed as a fabric to fabricate aprons [19]. For this purpose, the density and thickness of the shielding fabric were considered vital factors in the manufacturing process [20]. In this study, the pencil beam spray-coating method was used to disperse uniformly metal particles onto a shielding sheet, a fabric used for shielding clothing in medical institutions and to control the dispersion of the same metal particles in a mixed state with the polymer. The shielding fabric developed using the proposed technology was compared with that fabricated using the conventional calender method in terms of the particle dispersion and shielding performance. The calender method is a representative process utilized for mixing polymer materials to form sheets [21]. The calender process adversely affects the shielding performance owing to voids, pores, and cracks generated during manufacturing [22]. The feasibility of a flexible medical radiation-shielding sheet is presented based on shielding sheet process technology by increasing the dispersion and controlling the spacings between pairs of neighboring particles using a pencil beam spraying device.

## 2. Materials and Methods

The protective effect of radiation-shielding sheets reduces the intensity and dose of incident energy [23]. As shown in Equation (1), the radiation energy transmitted through the shielding sheet can be expressed as a product of the incident radiation fluence and dose function *R*(e) [24]. The radiation fluence is included owing to the interaction with the material when radiation penetrates the shielding sheet [25].

(1) $$R\left(D\right)=\int_{E}\emptyset \left(e\right)R\left(e\right)D\left(e\right)$$

The material composition influences the interaction inside the shielding sheet, depending on the effect of the shielding material. The shielding rate can be estimated by comparing the remaining strength of the beam (μ) with its initial strength (x) after it passes through the thickness *(*$I_{0}$) of the shielding sheet based on the linear attenuation coefficient (I), as shown in Equation (2) [26]. The mass attenuation coefficient ($\mu_{m}$) can be calculated according to Equation (3) using the linear attenuation coefficient (μ) according to the thickness (x) of the shielding sheet [27]. To enhance the efficiency of a medical radiation-shielding sheet when a composite material is utilized, Equation (4) can be defined by considering the product of the weight and mass attenuation coefficient of each material [28].

(2) $$I=I_{0}e^{-ux}$$

(3) $$\mu =\frac{\left(1n\frac{D_{0}}{D}\right)}{x},\;\;\mu_{m}=\frac{\mu }{\rho }$$

(4) $$\mu_{m}=\sum \omega_{i}(\frac{\mu }{\rho }{)}_{i}$$

**Figure 1:**
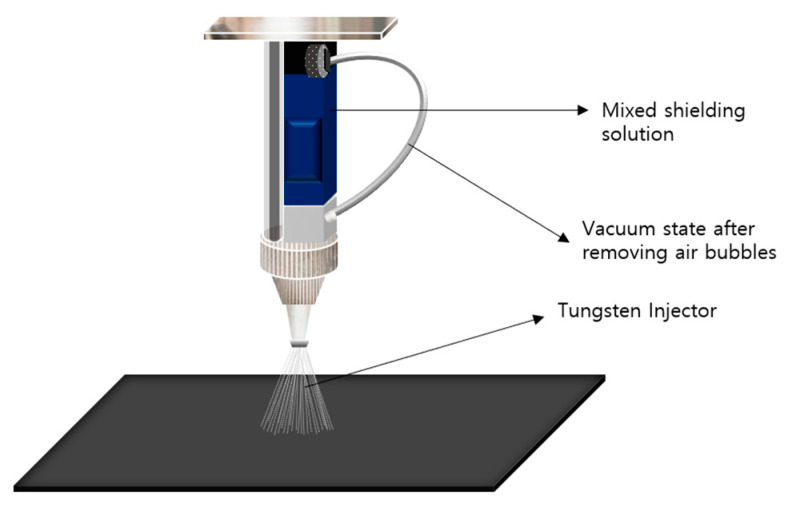
Spraying of the tungsten mixture solution.

**Figure 2:**
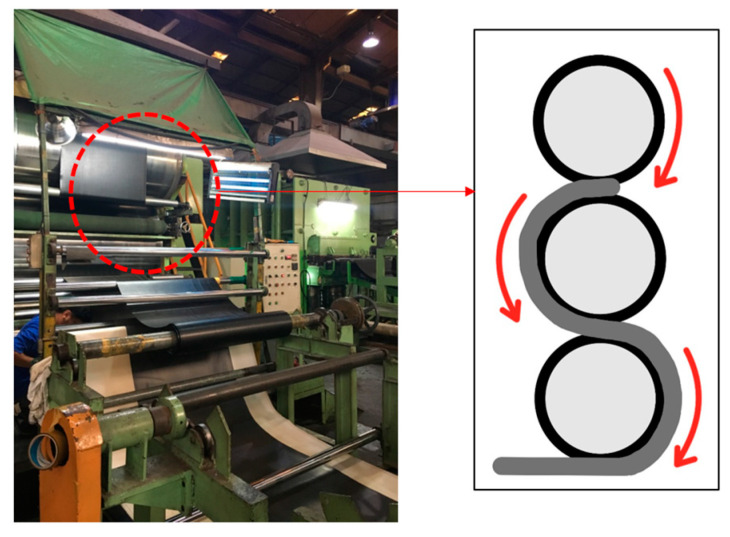
Roller compression applied during the tungsten sheet calender process.

Therefore, the arrangement of radiation-shielding materials within the same shielding sheet area reduces the number of voids and increases the density. Additionally, converting the incident energy into a form that increases the interaction probability is crucial [29]. This study used microtungsten particles (tungsten, W, 99.9%, <5 µm, NanGong XinDun Alloy Spraying Co. Ltd., Xingtai, China) and high-density PE (HDPE-S, molecular weight: 10,000–140,000, Songwon, Korea) as the shielding and base polymer materials, respectively. The tungsten powder was pulverized into particles with diameters ≤5 µm using ultrasonic grinding equipment and dried at 70 °C for 12 h before use [30]. Tungsten was coated with a polymer material and mixture for the shielding sheet. N-dimethylformamide (DMF, 99.5%, Daejung, Korea) was utilized as the solvent for polymer dissolution. Further, two solvents were used to prepare the shielding sheets. DMF (for polymer dissolution) and chloroform (95%, Duksan, Korea) were used as poor solvents to control the solvent volatilization rate. The casting solution was stirred at 5000 (rpm) revolutions/min to disperse the tungsten particles. The spinning solution was mixed with three tungsten contents (75, 80, and 85 wt%). As shown in Figure 1, the sheet was produced by pressing the surface several times after spray coating. Stagnation occurred in the injector when the amount of microsized tungsten exceeded 85 wt% during mixing; therefore, mixing was performed until the tungsten amount reached 85 wt%. As shown in Figure 2, a coating was produced with the same tungsten ratio throughout the calender process after mixing it with HDPE as a single material. Air bubbles were removed from the mixed material through defoaming, and this process technology was performed by focusing solely on the uniform dispersion of particles [31]. During the calendering process, the thickness was controlled by a multiroller compression method to form a sheet. Production in the form of sheets was achieved by removing a sufficient number of air bubbles and reducing the gaps between particles via stirring. However, the spray-coating method required stirring after spinning to prevent the occurrence of ratio differences during the spraying process due to the specific gravity of tungsten and the bubble removal process. Therefore, after the first spraying cycle, the mixture was mixed using an ultrasonic vibrator to ensure that the tungsten dispersed well in the solution. As the spray volume was low, one additional agitation was sufficient. The injection method involved spraying 50 cc at a speed of 10 mm/s; based on these settings, the thickness could be controlled. In this processing technology, the important height is the distance from the nonwoven fabric (at the bottom part) to the spraying point. When the distance is small, the gap between particles tends to be narrow, and when the distance is large, the gap between particles tends to be broad. Therefore, it was set to approximately 15 cm by fixing the spraying interval. The shielding performances of the shielding sheets (thicknesses: 0.48–0.54 mm) were comparatively evaluated.

**Figure 3:**
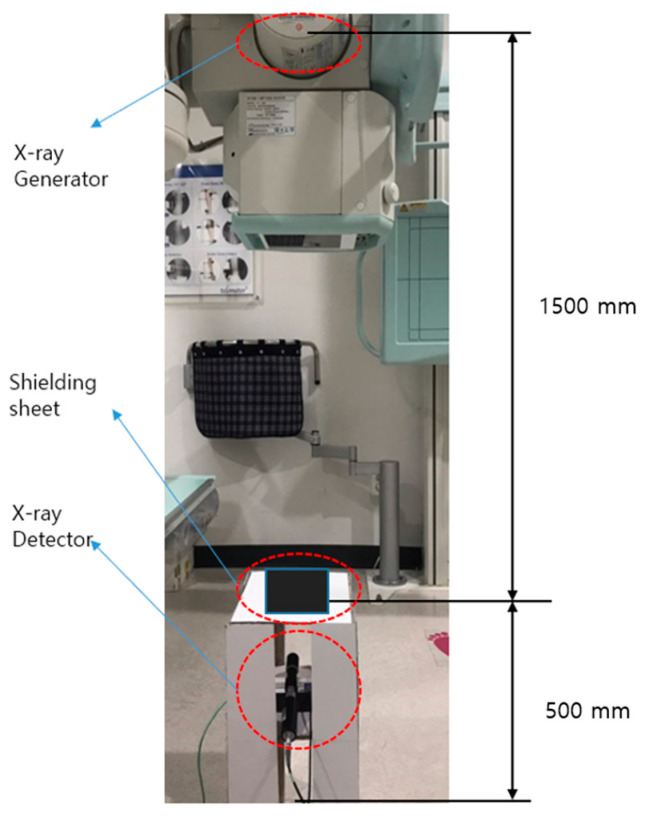
Photograph of the setup of the shielding performance evaluation method.

To confirm the distribution of tungsten particles across the shielding sheet, we performed scanning electron microscopy (SEM) at 15.0 kV × 10.0 k using a field-emission scanning electron microscope [32]. An X-ray generator (MOBIX-1000, Listem, Inchun-City, Republic of Korea, 2010) was employed to test the shielding performance. A radiation detection dosimeter was installed (see Figure 3) using an ion chamber ionization bath (Radcal 2186 (Accu-Dose), Radcal Co, 2020) device [33]. To convert the incident radiation used in this experiment from a single to an effective energy setting, the half-thickness was obtained using the attenuation coefficient law $\left(I=I_{0}e^{-\mu x}\right)$, from which the slope was calculated. Once the pre-absorption coefficient μ was obtained, the half-thickness was calculated according to the expression half-thickness = 0.693/μ [34]. For effective energy calculation, Hubbell’s mass absorption coefficient table was used to compute the effective energy that was equal to the single energy at the half-thickness value [35].

The shielding performances of the sheets fabricated with the two different manufacturing technologies were calculated using the shielding rate based on considerations of radiation protection efficiency [36]. In the experiment, the shielding rate of the radiation-shielding sheet was calculated using Equation (5), where e and $e_{0}$ are the radiation doses measured with and without a shielding sheet between the X-ray beam and detector, respectively.

(5) $$REF=\left(1-\frac{e}{e_{0}}\right)\times 100,$$

where REF is the shielding rate, $e_{0}$ is the incident exposure (μR), and e is the penetration dose (μR).

## 3. Results

Table 1 lists the general characteristics of the sheets manufactured via the pencil beam spray-coating and calender methods using a composite material mixed with PE and tungsten microparticles. The density was the most significant feature that exhibited the major differences between the two methods. In general, the higher the wt% and metal particle content, the smaller the difference; however, the spray-coating method mostly yielded values higher than those of the conventional calender method. The spray-coating method was expected to have a large contact area with air, which could result in the formation of many bubbles and voids; however, multiple coatings were performed, and the density was improved by converting the material into a sheet through a rolling process.

**Figure 4:**
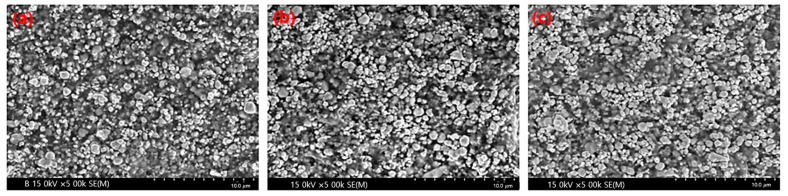
Cross-sectional image of the sheet produced using the pencil beam spray-coating method at various tungsten contents: (**a**) 75 wt%, (**b**) 80 wt%, and (**c**) 85 wt%.

**Figure 5:**
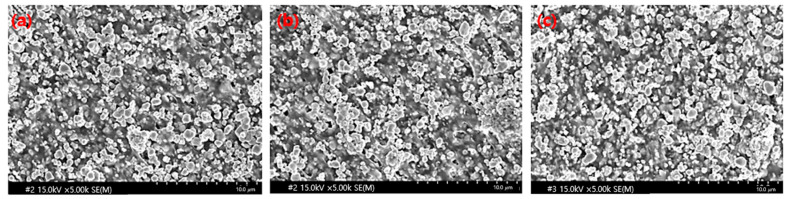
Cross-sectional images of sheets produced using the calender method at various tungsten contents: (**a**) 75 wt%, (**b**) 80 wt%, and (**c**) 85 wt%.

The sheets manufactured using the two process technologies were compared and analyzed using electron microscopy. As shown in Figure 4, minor differences in their densities were observed. The spacings between the tungsten particles were more densely controlled in the spray-coating method than in the general calender process. With the increase in the tungsten content, the gap between the particles became narrower, and the PE agglomeration effect became more prominent. In the calender method shown in Figure 5, the polymer material was coated around the tungsten particles to form a thick layer, and the same high-molecular weight was applied; however, the particles appeared large owing to the agglomeration phenomenon.

**Figure 6:**
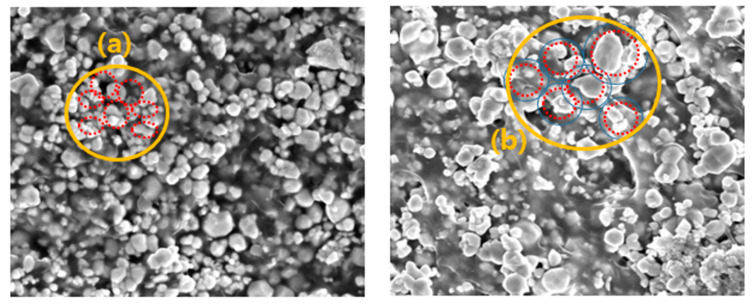
Comparative analysis of the coating type of polymer and shielding material: (**a**) pencil spray-coating and (**b**) calender methods.

The distances between the particles in the spray and calender methods were compared, as shown in Figure 6. Figure 6a,b shows the particle distributions of the sheets manufactured through the spray and calender methods, respectively. In Figure 6a, it can be seen that the tungsten particles separated and settled without being coated with the polymer. In contrast, in Figure 6b, the tungsten particles were coated with the polymer and agglomerated. It was assumed that this occurred during the stirring process (which lasted for 180 min), which took advantage of the narrowing of the interparticle spacing; however, agglomeration could also cause void generation.

**Figure 7:**
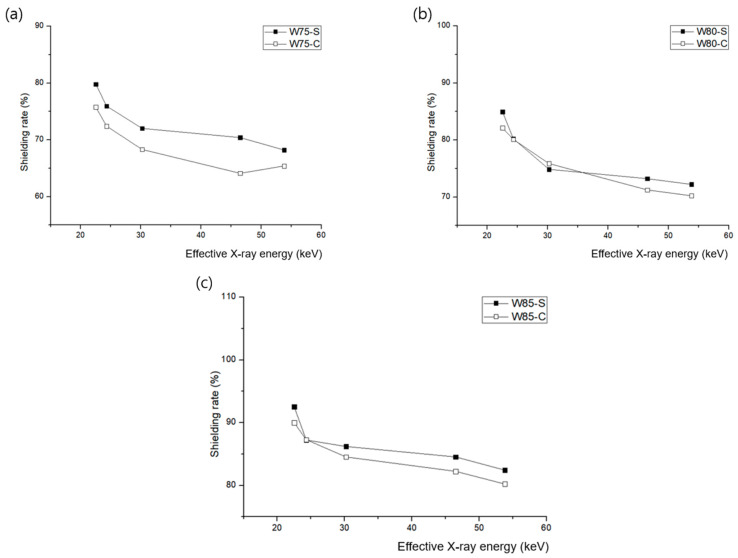
Comparison of the shielding performances according to the polymer coating type and shielding material used at various tungsten contents: (**a**) 75 wt%, (**b**) 80 wt%, and (**c**) 85 wt% (C: calender method, S: pencil spray method).

The shielding performances of the sheets manufactured using the two methods differed, as shown in Table 2 and Table 3. The shielding sheet manufactured using the spray-coating method exhibited a high-shielding rate in the low-effective energy area. The sheet manufactured through the calender method exhibited a similar pattern. The shielding performance data of the high-incident-energy and low-energy regions were as expected; however, a difference was observed in the middle-energy region, as shown in Figure 7. When the tungsten content was 80 wt%, a singularity was observed in which the two process technologies exhibited almost similar shielding performances. Thus, the spray-coating method was more effective when the metal particle content was reduced. When the standard lead equivalent was converted based on the high-tungsten content of 85 wt%, the solution spray method outcome corresponded to 0.243 mmPb, while the calender method outcome corresponded to 0.223 mmPb. It can be confirmed from the shielding ratio that there is a difference depending on the distribution state of the particles.

## 4. Discussion

In general, the shielding sheets used in medical institutions are manufactured taking into account the shielding performance. A basic design for radiation defense, such as a single or composite structure, is necessary to satisfy the conditions for this application [37]. A general shielding design was proposed for a composite structure by evaluating the shielding performance based on the material density. Composites mixed with other materials, such as polymers, are manufactured with the same thickness; however, their performances differ depending on the mixing technology and stirring method used. Thus, the composition and location of the shielding material particles directly affect the density, thereby influencing the interaction of the incident radiation [38]. To overcome this limitation, many researchers have conducted studies to exclude voids by combining single materials or reducing the particle size to nanoscale levels [39]. Although this method is effective, the economic feasibility, product commercialization, and marketability for mass production are limited. Therefore, this injection method demonstrates excellent feasibility for obtaining high efficiency at a low cost. The thickness and density of the shielding fabric for manufacturing radiation-shielding suits used in medical institutions can be controlled by reducing the air gaps using laminated structure shield designs in conjunction with the multiparticle dispersion method. Controlling the thickness and density to obtain lightweight shielding clothing is important to ensure user mobility.

Therefore, the primary aim of process technologies for shielding sheet manufacturing is to reduce the thickness and weight based on optimal metal particle dispersion [40]. Among the existing sheet manufacturing technologies, calender processing is a forming method in which raw materials are rolled between two or more rolls rotating in opposite directions; it is the most common cost-effective manufacturing process that enables mass production. However, controlling pores and bubbles through a repetitive process is necessary because the mixing ratio of metal particles in the primary shielding material is proportional to the shielding performance [41]. The proposed pencil beam spray-coating method does not increase the size by overlapping polymer chains on the surface of tungsten particles. A method of blocking voids, which causes the generation of pinholes, was proposed by narrowing the gap between the metal particles to increase the interfacial adhesion strength. In addition, bubbles generated by repeated injection methods can be reduced by adjusting the thickness using chemical and press compression methods. This method is similar to the design of a multilayered shielding sheet [42]. The performances of the two process technologies can be compared based on either the shielding rate or the standard lead equivalent. A lead equivalent of 0.25 mmPb is the design criterion for aprons, and our method could achieve a lead equivalent of 0.243 mmPb, which is considered appropriate for commercialization. The dispersion of the radiation-shielding material in the sheet directly affects the shielding performance. An improper dispersion design results in cracks, pores, and pinholes, thus reducing the shielding performance and causing problems in hardness and durability. The deterioration of miscibility with the polymer materials primarily obstructs the uniform dispersion of the shielding material; agglomeration is a representative example [43]. Various process technology parameters, such as temperature, agitation speed, and particle size, can affect the process output. Therefore, dispersion of the shielding material is possible only when the spacings between particles and the polymer concentrations are reduced [44]. To reduce the polymer concentration, this study proposed position fixing using direct spraying centered on metal particles that were designed and manufactured to fix the shielding material within the sheet area.

This study had some limitations. We attempted to apply the proposed process technology based on mass production; however, the sheet manufactured in the experiment used a single-spraying mechanism that required approximately 2 h for 1 m^2^ and involved the mixing of tungsten particles after a single-spraying cycle. The production time can be shortened by adding a single-line arrangement of injection devices and a self-stirring device, which will require extensive research in the future. For the manufacture of radiation-shielding clothing worn in medical institutions, a spraying method was proposed as a process technology to ensure flexibility by increasing the density and lowering the thickness to ensure mobility. The even dispersion of tungsten particles improved the shielding performance. These technologies are important for achieving effective shielding-suit manufacturing processes.

## 5. Conclusions

A pencil beam spraying method was developed to enhance the dispersion of a shielding material. Compared with the existing calender coating method, the degree of dispersion of the metal particles was improved and the shielding performance was enhanced by 2–4%. Therefore, the improved processing technology confirmed the effect of maintaining constant distances between metal particles, as shown in the cross-sectional SEM images of the manufactured sheets. This method is envisaged to be important for manufacturing shielding fabrics for medical-radiation-shielding suits.

## Figures and Tables

**Table 1:** General sheet characteristics for different coating methods.

Sample		Weight $\left(\mathrm{kg}/m^{2}\right)$	Thickness (mm)	Density $\left(g/{cm}^{3}\right)$
W75	Calender	1.084 ± 0.039	0.545 ± 0.011	2.019 ± 0.004
	Pencil spray	1.152 ± 0.039	0.532 ± 0.032	2.248 ± 0.012
W80	Calender	1.257 ± 0.052	0.525 ± 0.064	2.301 ± 0.025
	Pencil spray	1.324 ± 0.052	0.522 ± 0.015	2.321 ± 0.015
W85	Calender	1.402 ± 0.052	0.499 ± 0.011	2.524 ± 0.035
	Pencil spray	1.454 ± 0.033	0.488 ± 0.021	2.642 ± 0.021

**Table 2:** Shielding performance of sheets manufactured using the pencil beam spray-coating method.

Radiation Type	Effective X-ray Energy (keV)	Mean of Exposure (μR)				Shielding Rate (%)		
		Nothing	W75	W80	W85	W75	W80	W85
X-ray	22.5	198.92	40.162	29.957	14.839	79.81	84.94	92.54
	24.3	450.24	108.283	89.012	57.451	75.95	80.23	87.24
	30.2	904.56	253.186	227.497	124.739	72.01	74.85	86.21
	46.5	1524.12	450.987	408.312	235.629	70.41	73.21	84.54
	53.8	1874.25	595.824	520.854	329.118	68.21	72.21	82.44

**Table 3:** Shielding performance of sheets manufactured using the calender method.

Radiation Type	Effective X-ray Energy (keV)	Mean of Exposure (μR)				Shielding Rate (%)		
		Nothing	W75	W80	W85	W75	W80	W85
X-ray	22.5	198.92	48.198	35.527	19.872	75.77	82.14	90.01
	24.3	450.24	124.176	89.553	57.226	72.42	80.11	87.29
	30.2	904.56	286.655	218.270	139.845	68.31	75.87	84.54
	46.5	1524.12	547.007	438.337	270.684	64.11	71.24	82.24
	53.8	1874.25	648.303	558.339	370.914	65.41	70.21	80.21

## Data Availability

Not applicable.
